# Genetic structure of *Octopus minor* around Chinese waters as indicated by nuclear DNA variations (Mollusca, Cephalopoda)

**DOI:** 10.3897/zookeys.775.24258

**Published:** 2018-07-17

**Authors:** Faiz Muhammad, Zhen-ming Lü, Liqin Liu, Li Gong, Xun Du, Muhammad Shafi, Hubdar Ali Kaleri

**Affiliations:** 1 National Engineering Research Center of Marine Facilities Aquaculture, College of Marine Sciences and Technology, Zhejiang Ocean University; 2 Center of Excellence in Marine Biology, University of Karachi; 3 Lasbella University of Agriculture, Water and Marine Sciences; 4 Department of Animal Science and Aquaculture, Dalhousie University, Canada

**Keywords:** Cephalopoda, China Coast, Nuclear DNA, Octopuses

## Abstract

*Octopus minor* is an economically important resource commonly found in Chinese coastal waters. The nuclear gene (RD and ODH) approach of investigation has not reported in this species. Rhodopsin (RD) and octopine dehydrogenase (ODH) genes were used to elaborate the genetic structure collected from eight localities ranging from the northern to the southern coast of China. In total, 118 individuals for the RD gene and 108 for the ODH were sequenced. Overall (RD and ODH) genes resulted in high (0.741±0.032; 0.805±0.038) haplotype and low nucleotide (0.01261±0.00165; 0.00747±0.00086) diversity. Molecular variance displayed higher values among the populations and lower values within the population where the fixation index F_ST_ denoted 0.880 and 0.584 in RD and ODH genes respectively. The Dongshan population clustered separately in a phylogenetic tree as in the haplotype networking assessment. The current data suggests that the Dongshan population needs separate management.

## Introduction

The class Cephalopoda embraces animals which are exclusively marine inhabitants. They have immense commercial and ecological significance, including a profound contribution as a source of protein for humans. The cephalopod has lobed and folded brain like that of vertebrates and are ingenious, migrant and largest of all molluscs (Mather and Kuba 2013; Cheng et al. 2013; Larson et al. 2015) and it is sensitive to environmental factors (Emery et al. 2016; Wang and Zheng 2017

Among cephalopods, the octopuses contribute 33% to the existing cephalopod assembly. Three hundred species of octopus are thought to exist along the coastal waters of Korea, China, and Japan (Roper et al. 1984; Norman and Sweeney 1997; Kang et al. 2012). One hundred thirty-four species are reported only in Chinese waters including the profit-making species like *Octopus minor*, *Amphioctopus fangsiao*, and *Cistopus chinensis* (Lu et al. 2012).

Previously, mtDNA molecular markers were popular because of their high mutation rate, maternal inheritance, and non-recombination (Vaseeharan et al. 2013). In the last decade, the nuclear DNA markers have been widely used for various investigations including identification, population genetics, comparisons between wild and captive populations, demographic evaluations, and rehabilitation projects (Chauhan and Rajiv 2010).

Carlini et al. (2000) was first who used nuclear DNA (Actin) for phylogenetic analysis of coleoid species subsequently other authors continued nuclear approach of investigation such as, (Warnke et al. 2003; Lindgren et al. 2004; Strugnell et al. 2005). The nuclear gene provides more information than mtDNA genes (Graybeal 1994). The rhodopsin and octopine dehydrogenase genes were used for phylogenetic and phylogeographic analysis of octopuses (Strugnell and Nishiguchi 2007; Toussaint et al. 2012). However, meagre information is available on population genetics of octopuses using RD and ODH genes. The RD and ODH genes are less complex and can provide better results than 18S rDNA. Our present study aims to focus on collecting fundamental information about the population structure of this species using nuclear genes.

## Materials and methods

**Figure 1.**
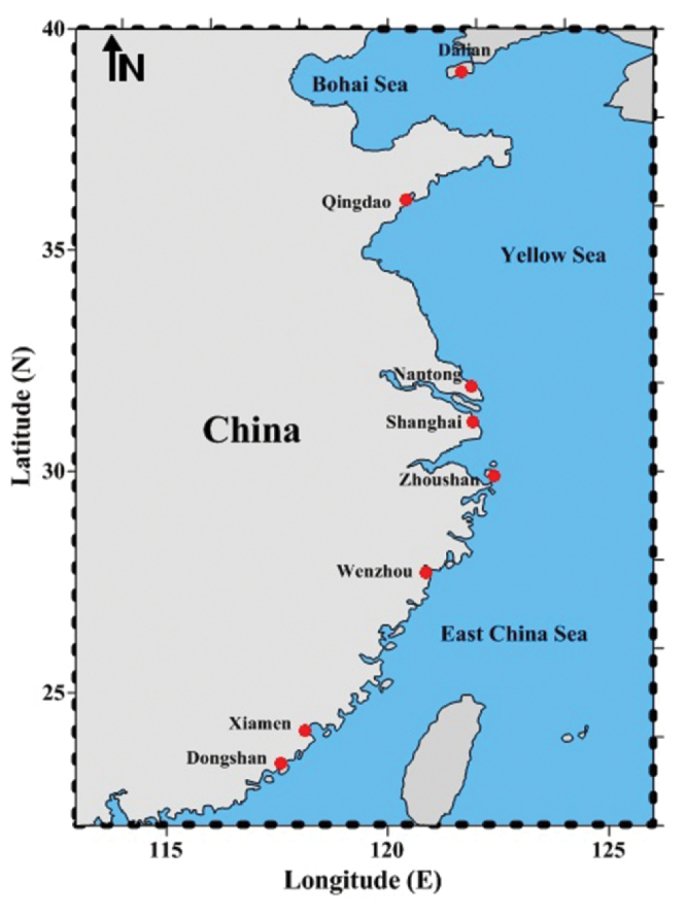
Map showing the collection locations.

Samples were collected from eight locations (Fig. 1). Thereafter, they were preserved in 95% ethanol and transported to the laboratory. The total genomic DNA was isolated from muscle tissues using a standard protocol. Target genes were amplified by PCR using primers (Table 1). The total 25 µl PCR mixture included (DNA template 1.25 µl, each primer 1.25 µl, ES Taq 12.5 µl, and 8.75 µl water). Thermocycler conditions were as follows: denaturation at 94 °C for 5 min, 35 cycles 94 °C for 30 s, annealing at 55 °C for 30 s, extension at 72 °C for 30s, and the final extension at 72 °C for 7 min. Electrophoresis was performed on a 1.2% agarose gel and was sequenced using the same oligonucleotide primers. Sequences were aligned using MEGA 6 software (Tamura et al. 2013). Analysis of genetic differentiation, AMOVA, molecular diversity indices, genetic differentiation values, and F_ST_ values were determined with the ARLEQUIN software (Excoffier and Lischer 2010). Calculations of gene flow (N_m_) were performed using formula *N_m_*= (1-F_ST_)/2F_ST_. Haplotype and nucleotide diversity were estimated using DnaSP (Librado and Rozas 2009). The neighbor joining tree was constructed to check the genetic relationship between populations using MEGA 6 (Tamura et al. 2013). The haplotype networking was created using NETWORK software version 5.0.0.1 (Bandelt et al. 1999).

**Table 1.** Oligonucleotide primers used for the amplification of nuclear rhodopsin and octopine dehydrogenase genes in *Octopus minor*.

Gene	Primer and sequence (5’-3’)	Tm (C)	size (bp)	Reference
Rhodopsin	RDF 5’-GCTTTCCTCATGATTATC-3’	50	653	Toussaint et al. 2012
	RDR 5’-TTCTCCATCATTGCCATC-3’			
Octopine dehydrogenase	ODHF 5’-AAATCCCGACCAAACATG-3’	50	618	Toussaint et al. 2012
	ODHR 5’-GTTAAGTTTGTACCAGTC-3’			

## Results

**Figure 2.**
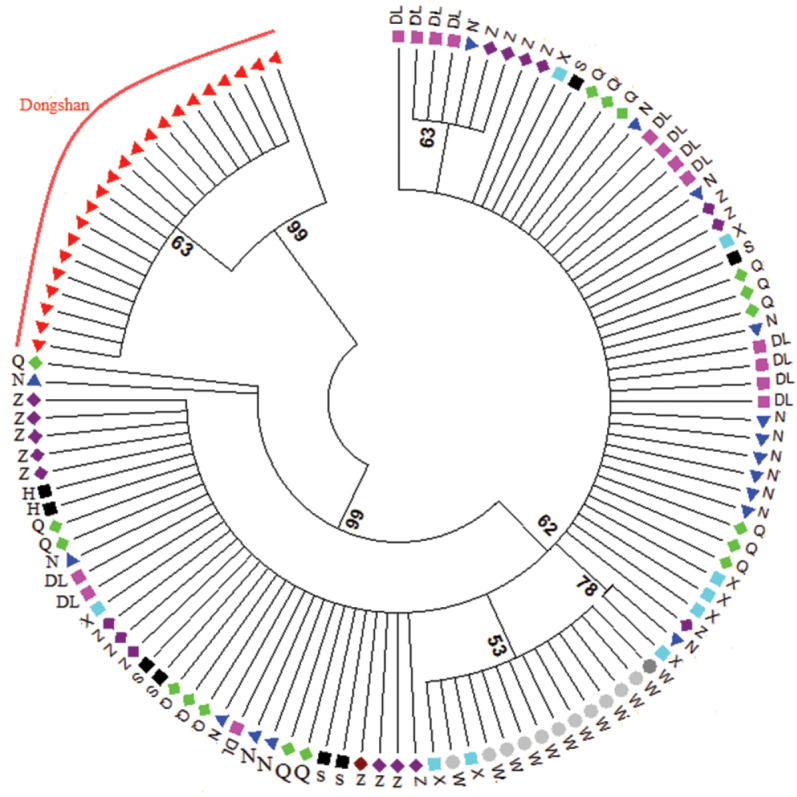
Neighbour-joining phylogenetic tree constructed based on RD gene sequences. Key: DL = Dalian, N = Nantong, Q = Qingdao, S = Shanghai, W = Wenzhou, X = Xiamen, Z = Zhoushan.

**Figure 3.**
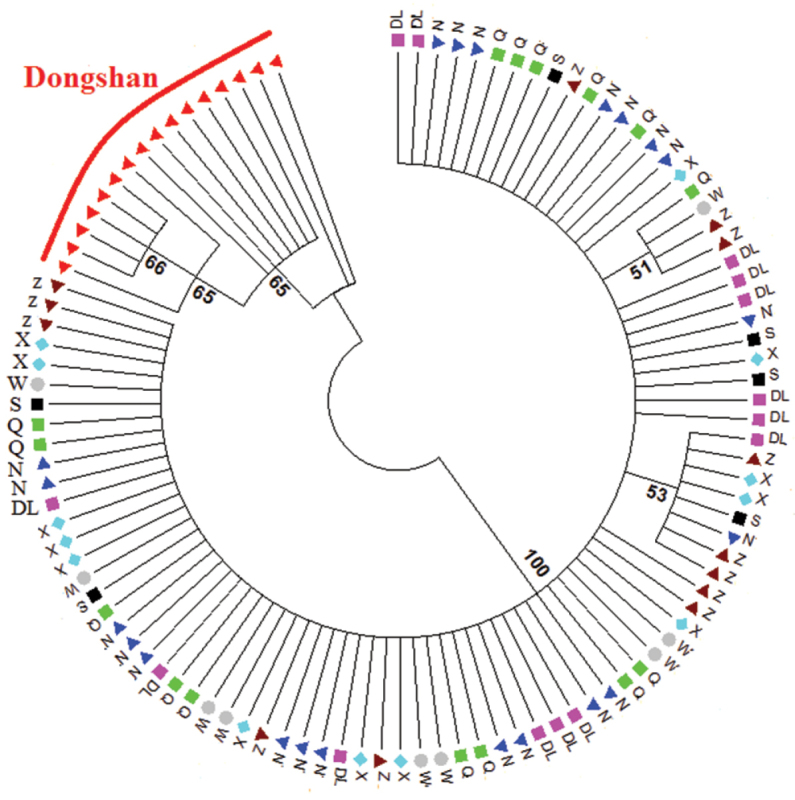
Neighbour-joining tree constructed based on the ODH gene. Key: DL = Dalian, N = Nantong, Q = Qingdao, S = Shanghai, W = Wenzhou, X = Xiamen, Z = Zhoushan.

**Figure 4.**
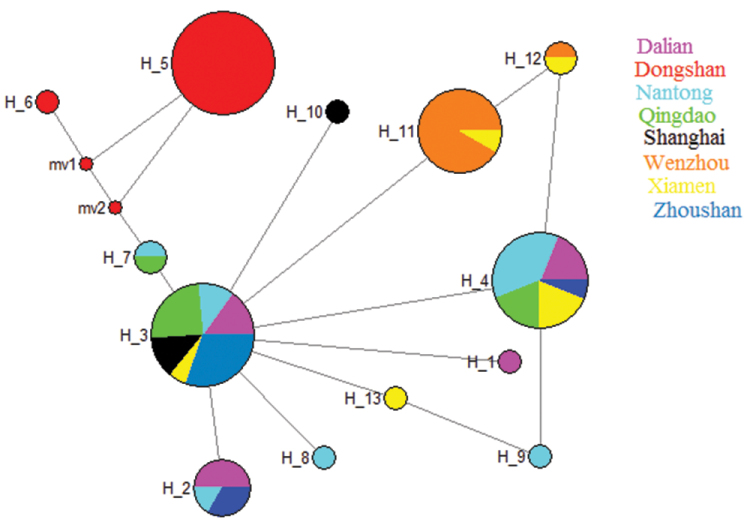
Median-joining networking drawn based on RD gene haplotypes. Colours represent the corresponding population frequencies. Key: Dalian; Dongshan; Nantong; Qingdao; Shanghai; Wenzhou; Xiamen; Zhoushan.

**Figure 5.**
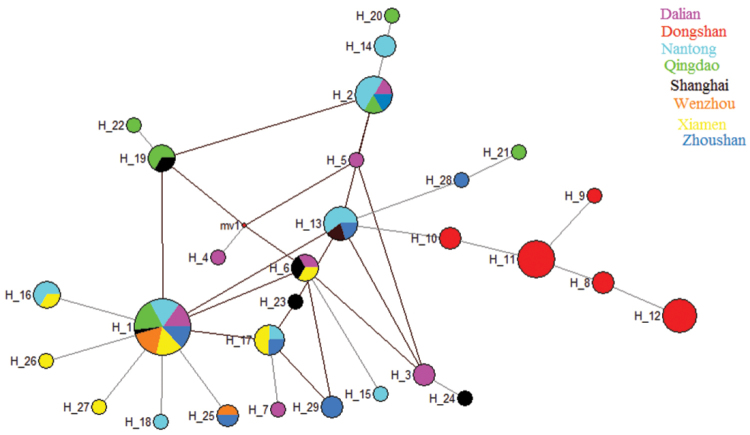
Median-joining networking drawn based on ODH gene haplotypes. Colours represent the corresponding population frequencies. Key: Dalian; Dongshan; Nantong; Qingdao; Shanghai; Wenzhou; Xiamen; Zhoushan.

*Rhodopsin Gene (RD)*: A fragment of 637 bp of RD was sequenced from 118 individuals. RD gene showed 33 polymorphic sites and 13 haplotypes. Haplotype diversity (HD) ranged from 0.090 to 0.833, nucleotide diversity (π) remained in the lower range (0.00027–0.001), where the average number of differences (k) ranged from 0.166 to 1.388 (Table 2). Molecular variance revealed a higher percentage among the populations (88.04%) while lower values were retrieved within (11.96%) populations; fixation index F_ST_ was 0.880 (Table 3). The pairwise F_ST_ values ranged from 0.014 to 0.989. The lowest gene flow was observed in Dongshan population (Table 4). The values of Tajima’s D tests generally showed negative values with exception to Xiamen population. The Wenzhou and Dongshan populations were statistically significant (P<0.05). Fu’s Fs values were positive and statistically non-significant in all populations (Table 6). The phylogenetic analysis of eight populations separated Dongshan population with 99% bootstrap support (Fig. 2). The networking analysis revealed that haplotype three (Hap 3) was shared by six populations (Dalian, Nantong, Zhoushan, Qingdao, Shanghai, and Xiamen), Hap 4 was contributed by five populations (Dalain, Nantong, Qingdao, Xiamen, Zhoushan), Hap 2 shared between Dalian, Zhoushan, and Nantong, while Hap 7 appeared in two populations (Qingdao, Nantong), Hap 11 and Hap 12 were present in the Wenzhou and Xiamen populations, and Hap 5 and Hap 6 were independently represented in the Dongshan population (Fig. 4). *Octopine dehydrogenase (ODH)*: A fragment of 597 bp was sequenced from 108 individuals, revealed 27 polymorphic sites and 29 haplotypes. Haplotype diversity remained higher (0.222–1.000) than nucleotide diversity (π) (0.001–0.004). The average number of differences (k) ranged from 0.444 to 2.733 (Table 2). Molecular variance was revealed to be higher among the populations (58.44%) while lower within a population (41.56%). Fixation index (F_ST_) was determined as 0.584 (Table 3). The pairwise F_ST_ values ranged from 0.018 to 0.925. The lowest gene flow was observed in Dongshan population whereas highest N_m_ values were shown between Wenzhou and Xiamen populations (Table 5). Tajima’s D showed negative values except in Dongshan, Shanghai, and Zhoushan populations whereas only Xiamen population was statistically significant (P<0.05). Fu’s Fs presented negative values except Dongshan, Qingdao, and Wenzhou populations while only Shanghai and Xiamen populations were statistically significant (Table 6). The neighbour-joining phylogenetic tree described two distinct clades where Dongshan population clustered separately with 100% bootstrap support (Fig. 3). The median joining network analysis described that Hap1 shared by seven populations followed by Hap2, which appeared in four populations, namely Dalian, Nantong, Qingdao, and Zhoushan. Hap 13 contributed by three populations (Dalian, Shanghai, and Zhoushan) similarly Hap 6 appeared in Dalian, Shanghai and Xiamen populations, Hap 17 shared by Nantong, Zhoushan, and Xiamen populations, Hap 16 shared by Nantong and Xiamen, Hap 19 appeared in Qingdao and Shanghai populations while Hap, 25 shared between Zhoushan and Wenzhou populations. Hap 8-12 were independently representing Dongshan population (Fig. 5).

**Table 2.** Genetic diversity parameters for RD and ODH sequences in *Octopus minor*.

Gene	Population	Number of segregating sites	Number of ha2lotypes	Haplotype diversity (Hd)	Nucleotide diversity (π)	Average number of differences (k)
Rhodopsin (RD)	Dalian	3	4	0.676	0.001	0.819
	Dongshan	4	2	0.090	0.001	0.363
	Nantang	6	6	0.760	0.001	1.150
	Qingdao	2	2	0.404	0.001	0.426
	Shanghai	1	2	0.250	0.00040	0.250
	Wenzhou	1	2	0.166	0.00027	0.166
	Xiamen	4	5	0.833	0.002	1.388
	Zhoushan	2	3	0.292	0.0004	0.304
Octopine dehydrogenease (ODH)	Dalian	7	7	0.758	0.003	2.164
	Dongshan	4	5	0.775	0.002	1.341
	Nantang	8	8	0.828	0.003	2.076
	Qingdao	6	6	0.647	0.003	1.800
	Shanghai	6	6	1.000	0.004	2.733
	Wenzhou	2	2	0.222	0.001	0.444
	Xiamen	5	6	0.717	0.001	0.897
	Zhoushan	7	7	0.794	0.003	2.025

**Table 3.** Analysis of molecular variance of Octopus *minor* using RDand ODH.

Gene	Source of variation	df	Sum of squares	variance component	Percentage (%)
RD	Among population	8	573.404	5.57703 Va	88.04
	Within population	110	83.334	0.75758 Vb	11.96
	Total	118	656.737	6.33461	
	Fixation Index FST:	**0.880**			
ODH	Among population	8	174.799	1.79694 Va	58.44
	Within population	99	126.490	1.27768 Vb	41.56
	Total	107	301.290	3.07462	
	Fixation Index FST:	**0.584**			

**Table 4.** Pairwise F_ST_ below diagonal and gene flow (N_m_) values above diagonal RD gene.

	Dalian	Dongshan	Nantong	Qingdao	Shanghai	Wenzhou	Xiamen	Zhoushan
Dalian	–	0.012	15.125	35.214	4.654	0.071	7.436	∞
Dongshan	0.975	–	0.016	0.008	0.006	0.005	0.018	0.007
Nantong	0.032	0.968	–	1.112	0.2	0.298	∞	2.996
Qingdao	0.014	0.983	0.043	–	7.696	0.149	7.564	19.5
Shanghai	0.097	0.987	0.200	0.061	–	0.103	2.104	23.309
Wenzhou	0.875	0.989	0.626	0.770	0.828	–	0.575	0.118
Xiamen	0.063	0.964	∞	0.062	0.192	0.465	–	0.002
Zhoushan	∞	0.986	0.143	0.025	0.021	0.809	0.154	–

**Table 5.** Pairwise F_ST_ below diagonal and gene flow (N_m_) values above diagonal ODH gene.

	Dalian	Dongshan	Nantong	Qingdao	Shanghai	Wenzhou	Xiamen	Zhoushan
Dalian	-	0.086	0.884	14.2	∞	2.530	3.632	∞
Dongshan	0.853	-	0.086	0.075	0.107	0.040	0.050	0.082
Nantong	0.018	0.852	-	∞	∞	1.836	2.441	17.357
Qingdao	0.034	0.869	∞	-	9.303	3.885	4.261	23.309
Shanghai	∞	0.823	∞	0.051	-	1.702	2.406	∞
Wenzhou	0.165	0.925	0.214	0.114	0.227	-	25.815	6.852
Xiamen	0.121	0.908	0.170	0.105	0.172	0.019	-	12.32
Zhoushan	∞	0.859	0.028	0.021	∞	0.068	0.039	-

**Table 6.** Tajima’s D and Fu’s FS tests, corresponding *p* value for eight population of *O. minor*.

Gene	Population	Tajima’s D		Fu’s Fs	
		D	*p*	Fs	*p*
RD	Dalian	-0.260	0.449	1.447	0.805
	Dongshan	-2.139	0.001	3.781	0.939
	Nantang	-0.390	0.385	0.017	0.513
	Qingdao	-0.416	0.405	1.360	0.789
	Shanghai	-1.054	0.213	1.414	0.688
	Wenzhou	-1.629	0.017	1.558	0.752
	Xiamen	0.061	0.533	0.087	0.518
	Zhoushan	-1.125	0.167	0.934	0.666
ODH	Dalian	-0.455	0.335	-0.66426	0.345
	Dongshan	0.353	0.694	0.45027	0.612
	Nantang	-0.220	0.445	-0.77911	0.367
	Qingdao	-0.031	0.526	0.00070	0.532
	Shanghai	0.665	0.741	-2.73435	0.021
	Wenzhou	-1.512	0.057	2.30182	0.843
	Xiamen	-1.579	0.027	-2.26025	0.022
	Zhoushan	0.579	0.300	-0.95837	0.265

## Discussion

*Octopus minor* is a bottom-dwelling inhabitant and its migration is limited. The dispersal capacity has immense influence on population genetics. Tag- recapture investigations of *O. vulgaris* reported to be restricted within one km from the point of release (Melis et al. 2018 and references therein). Conversely, dispersal range of *O. minor* juveniles and adults is not precisely known. The basic information of genetic variation and population structure is valuable for stocking, fisheries management, and conservation (Feng et al. 2017). Several divergent forces cause genetic differentiation, including geographic isolation, current and life history characteristics (Gao et al. 2016). In fact, oceanic processes are complex and a single reason cannot be claimed as source of divergent force. There are many islands and gulfs in China’s sea, which can contribute to the gene flow complications of the populations (Gao et al. 2016). Earlier several studies were under taken including complete mitochondrial genome of this species (Cheng et al. 2012). Previous investigations on population genetics of *O. minor* present subtle to significant differences (Gao et al. 2016; Yang et al. 2015; Lü et al. 2013; Kang et al. 2012; Xu et al. 2011; Sun et al. 2010; Li et al. 2010). Our present analysis of two nuclear DNA gene sequences in *O. minor* collected from eight locations imparted variation. The haplotype diversity of RD gene ranged (0.090–0.833), the higher haplotype diversity was noted in Xiamen population and lowest haplotype diversity showed in Dongshan population. The ODH gene haplotype diversity was higher in Shanghai population (1.000) and was lowest in Wenzhou population (0.222). Comparative studies of Kang et al. (2012) between Korean and Chinese populations showed less haplotype diversity in Korean population than Chinese populations. However, only three Chinese populations were sampled (Dalian, Tianjin, and Rongcheng) from Bohai and Yellow seas, which showed 0.43 to 0.64 values of haplotype diversity. This result is in line with present study. Chang et al. (2010) reported higher haplotype diversity in Lianyungang (0.934) and lowest (0.342) in Xiamen population but RD and ODH gene showed higher values (0.833; 0.717) in Xiamen population. Yang et al. (2015) included five populations and reported that Qingdao has high diversity and more diverse than others but present research described high diversity of all northern populations (Table 2). The AMOVA results denoted higher values among populations and lower values within populations, where the fixation index F_ST_ was much higher. Yang et al. (2015) reported lower AMOVA values (10.88%) between populations and higher values (89.12%) within population, which is discordant. Higher F_ST_ values indicate a lower level of gene flow (N_m_) and higher genetic differentiation among populations (Hedrick and Goodnight 2005; Ye et al. 2015). The lowest gene flow between Dongshan with other counterpart populations was observed. F_ST_ value of 0.05 is considered to indicate negligible genetic differentiation, while a value greater than 0.25 demonstrate high genetic differentiation within the analyzed population (Weir and Cockerham 1996). Based on this standard, the results obtained in this study showed high differentiation between Dongshan and other counterpart populations (Tables 4, 5).

The Tajima’s D analysis of the RD gene showed negative values except for the Xiamen population, unlike to the results of the CO1 studies (Chang et al. 2010). The ODH gene revealed negative values for Tajima’s D except for three populations, Shanghai, Zhoushan, and Dongshan. Results are similar to mtDNA COI investigations (Chang et al. 2010) where the Zhoushan population had positive values; Shanghai and Nantong populations were not included in COI studies. The RD gene revealed 13 haplotypes; among them Hap 3 was common, shared by 6 populations. None of the haplotypes of the Dongshan population was shared by other counterpart populations with similar COII and Cytb investigations (Lü et al. 2013; Li et al. 2013). Nevertheless, it differs with COII and Cytb (Lü et al. 2013; Li et al, 2013) where Wenzhou, Zhoushan, and Wenzhou, Xiamen populations remained isolated populations respectively. The ODH gene described 29 haplotypes among which Hap1 remained common, which was shared by seven populations. It is consistent with Lü et al. (2013) and Li et al. (2013) with respect to Dongshan population whereas differ with reference to Wenzhou and Xiamen populations. Studies of Kang et al. (2012) described sharing of one haplotype between Chinese and Korean populations. The neighbour-joining phylogenetic tree of both the genes distinctly clustered Dongshan population separately as in Cyt b, and COII studies (Li et al. 2013; Lü et al. 2013). However, COII unveiled Wenzhou and Zhoushan populations as separate clade (Lü et al. 2013); similarly, 16S rRNA showed the Xiamen population as a separate clade (Li et al. 2010). Chang et al. (2010) reported Wenzhou and Xiamen populations as separate clade. Kang et al. (2012) investigated three clades where only one Chinese population (Dalian) parted as sub-clade along with Korean populations. Yang et al. (2015) using AFLP and Sun et al. (2010) using CO1 reported two clades, which is inconsistent with present study. The discrepancy of data is consequences of various unknown oceanic process, genetic markers used and range of sampling locations (Lü et al. 2013). To understand the data variation of *O. minor* along the Chinese coast, it is imperative to study the larval and adult dispersal range along with seasonal oceanic process during ontogenesis. Present data does not support the isolation by distance (IBD) because the geographic coastal distance between Dongshan and Xiamen is only 157 KM while the gene flow between them is meagre as shown by RD and ODH (0.018; 0.050), whereas the coastal distance between Dalian and Xiamen is approximately 3700 Km but the gene flow is higher (7.436; 3.632). Lü et al. (2013) was also noted gene flow discordance but the concrete reason is unknown. Melis et al. (2018) emphasized the causes of meagre larval dispersal in *O. vulgaris* including high mortality, philopatric behaviour of larvae and potential cryptic berries. Nevertheless, the above-mentioned factors are difficult to be disentangled, furthermore, oceanic fronts (temperature, salinity, density, turbidity, nutrients, velocity), upwelling and current systems can also influence the larval dispersal (Melis et al. 2018). It has long been entrenched that genetic structure of populations affected by glacier activities where sea level encountered climatic fluctuations during the Pleistocene period and caused gene flow restrictions in marine organisms (Imbrie et al. 1992). However, relatively high haplotype diversity reported in freshwater fish inhabiting non-glaciated regions or temperate regions (Bernatchez and Wilson 1998; Tan et al. 2015). Most of the studies related to *O. minor* gave high haplotype diversity in this region, including the present study.

Our present investigation has significant implications for conservation and favourable management of *O. minor* along the Chinese coast and it also shed light on the need for separate management of the Dongshan population: once an evolutionary lineage is lost, there is no possibility to be recover it (Mortiz 2002). We also recommend infield ontogenetic studies and larval migration ranges observations along with physical oceanographic parameters in future studies to understand the population genetics data inconsistency in *O. minor* reported along Chinese coastal waters.
